# The cut-off values of surrogate measures for insulin resistance in the Korean population according to the Korean Genome and Epidemiology Study (KOGES)

**DOI:** 10.1371/journal.pone.0206994

**Published:** 2018-11-12

**Authors:** Bongyoung Kim, Hyun Young Choi, Wonhee Kim, Chiwon Ahn, Juncheol Lee, Jae Guk Kim, Jihoon Kim, Hyungoo Shin, Jae Myung Yu, Shinje Moon

**Affiliations:** 1 Department of Internal Medicine, Hanyang University College of Medicine, Seoul, Republic of Korea; 2 Department of Emergency Medicine, Hallym University College of Medicine, Chuncheon, Republic of Korea; 3 Department of Emergency Medicine, Armed Force Yangju Hospital, Yangju, Republic of Korea; 4 Department of Emergency Medicine, Hanyang University College of Medicine, Seoul, Republic of Korea; 5 Department of Thoracic and Cardiovascular Surgery, Hallym University College of Medicine, Chuncheon, Republic of Korea; 6 Department of Emergency Medicine, Hanyang University College of Medicine, Hanyang University Guri Hospital, Guri, Republic of Korea; 7 Department of Internal Medicine, Hallym University College of Medicine, Seoul, Korea; Erasmus MC, NETHERLANDS

## Abstract

**Objective:**

The current methods available for determining insulin resistance are complicated; hence, they are only applicable to small-scale studies. Therefore, this study aimed to classify the characteristics of surrogate measures for insulin resistance and establish valid cut-off values for predicting the development of type 2 diabetes mellitus (DM) in Korean populations.

**Methods:**

This prospective study included 7,643 participants aged 40–69 years from the Ansung-Ansan cohort database (2001–2012). Four surrogate measures, namely homeostasis model assessment-insulin resistance (HOMA-IR), visceral adiposity index (VAI), lipid accumulation product (LAP), and triglycerides and glucose (TyG) index, were analyzed. We analyzed each measure using receiver operating characteristic (ROC) curve for the development of type 2 DM. The cut-off value was determined as the value with the highest Youden index score in the specificity dominant area.

**Results:**

The area under the curve (AUC) was 0.566 (95% confidence interval [CI], 0.548–0.583) for HOMA-IR, 0.622 (95% CI, 0.605–0.639) for VAI, 0.642 (95% CI, 0.625–0.658) for LAP, and 0.672 (95% CI, 0.656–0.687) for TyG index. The AUC of TyG index was significantly higher than that of HOMA-IR, VAI, and LAP (p < 0.001). The cut-off value was 2.54 (sensitivity 36.8%; specificity 73.1%; hazard ratio [HR], 1.41, 95% CI, 1.25–1.59) for HOMA-IR, 2.54 (sensitivity 50.4%; specificity 68.8%; HR, 1.75, 95% CI, 1.55–1.96) for VAI, 36.6 (sensitivity 59.2%; specificity 63.9%; HR, 1.87, 95% CI, 1.64–2.14) for LAP, and 4.69 (sensitivity 62.1%; specificity 63.1%; HR, 2.17, 95% CI, 1.92–2.45) for TyG index.

**Conclusions:**

The TyG index was a better predictor for DM than HOMA-IR. VAI and LAP showed the modest predictability for DM. The TyG index could be a useful supplementary method for identifying individuals at risk for insulin resistance and DM development.

## Introduction

Variable degrees of insulin resistance and impaired insulin secretion are major pathophysiological characteristics of type 2 diabetes mellitus (DM) [1]. Insulin resistance is characterized by a reduced physiological response of target tissues to normal levels of insulin and results in decreased glucose utilization in muscle and fat, as well as increased gluconeogenesis in the liver [2–4]. Understanding the contribution of insulin resistance to the pathogenesis of type 2 DM is important for establishing preventive measures and determining optimal therapeutic approaches. Unfortunately, the current methods (e.g., pancreatic suppression test, hyperinsulinemic-euglycemic [HIEG] clamp technique, and minimal model approximation of the metabolism of glucose [MMAMG]) available for determining insulin resistance are complicated, invasive, and expensive; hence, they are only applicable to small-scale studies [5–8]. Instead, indirect indices, such as homeostasis model assessment-insulin resistance (HOMA-IR), visceral adiposity index (VAI), lipid accumulation product (LAP), or triglycerides and glucose (TyG) index, are widely accepted for epidemiological or clinical studies because of their technical simplicity [9, 10, 11]. However, valid cut-off values of the indices used in predicting DM have not been fully evaluated yet. Hence, this study aimed to determine the characteristics of surrogate measures for insulin resistance in Korean populations and establish valid cut-off values for predicting DM.

## Materials and methods

### Study populations

The Ansung-Ansan cohort study is an ongoing prospective study that started in 2001 with support from the National Genome Research Institute in Korea’s Center for Disease Control and Prevention. Detailed information on the study design and procedures is available in a previous report [12].

**Fig 1:**
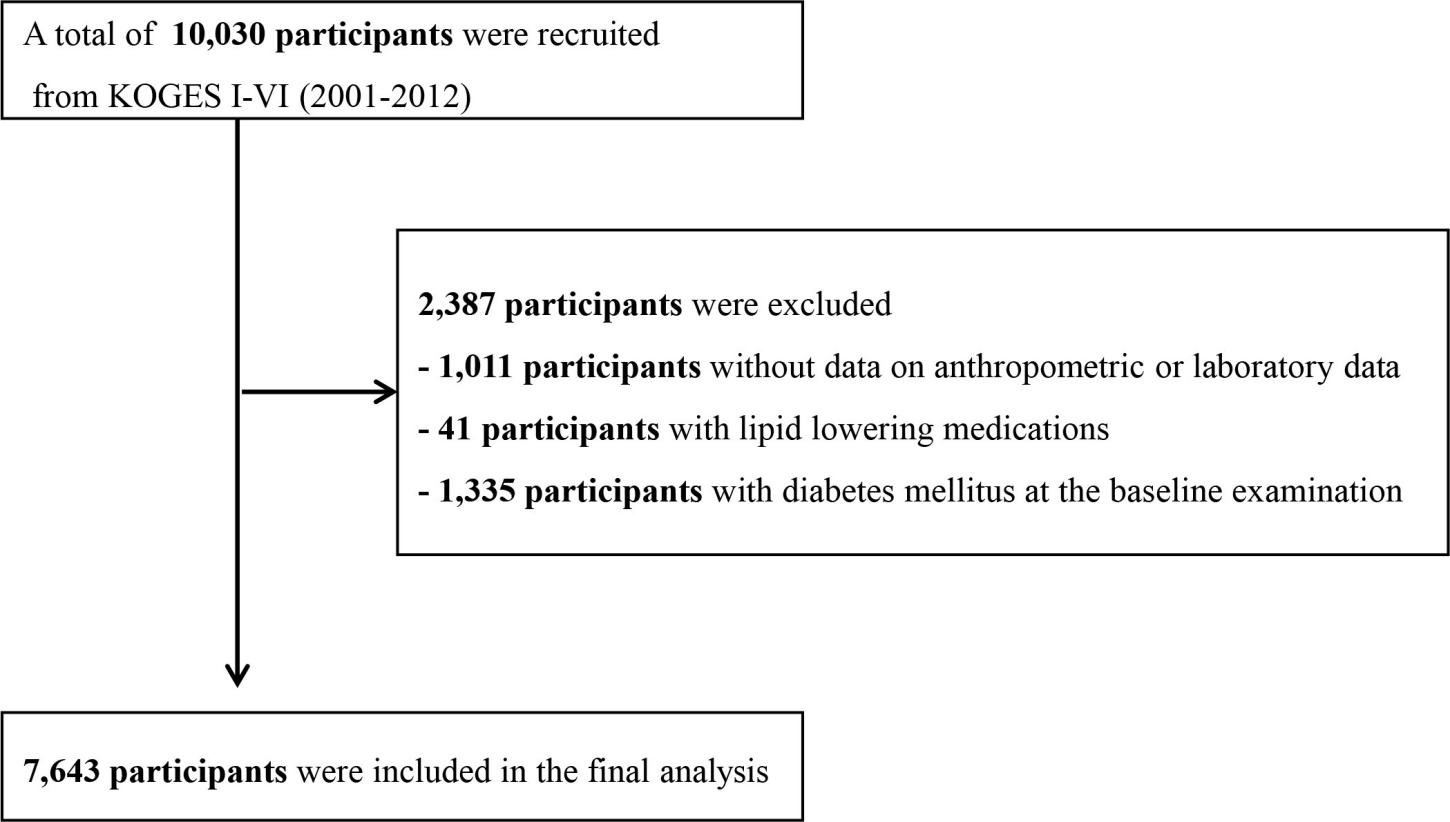
Flowchart showing the final selection. KOGES, The Korean Genome and Epidemiology study.

A population-based sample of male and female Koreans aged 40–69 years were enrolled from the following two sites: Ansung, which is a rural community with approximately 190,000 residents and Ansan, which is a rural community with approximately 693,000 residents [13]. A total of 10,038 participants (5,018 from Ansung and 5,020 from Ansan) underwent a baseline health examination at the Ajou University Medical Center and the Korea University Ansan Hospital from June 2001 to January 2003. Follow-up examinations were conducted biennially. Data from the baseline survey and five subsequent surveys (I-VI: 2001–2012) were analyzed in the present study. We excluded the following participants: those with incomplete data, those with lipid lowering medications and those with a clinical history of DM at the baseline examination (Fig 1). In total, 7,643 participants were eligible for this study.

### Clinical and laboratory measurements

Waist circumference was measured at the end of normal expiration using flexible tape at the narrowest point between the lowest border of the rib cage and the uppermost lateral border of the iliac crest. Height and body weight were measured to the nearest 0.1 cm and 0.2 kg, respectively. Blood pressure was measured in the sitting position after at least 5 minutes of rest. Blood samples were obtained after an overnight fast of at least 8 hours, and biochemical assays, including plasma glucose, total cholesterol, triglycerides, and high-density lipoprotein cholesterol (HDL-C), were measured using the ADVIA 1650 chemistry analyzer (Bayer HealthCare Ltd., Tarrytown, NY, USA). Hemoglobin A1c (HbA1c) level was measured using high-performance liquid chromatography (Variant II; BioRad Laboratories, Hercules, CA, USA).

### Definitions

A patient was deemed to have DM if he/she had at least one of the following conditions: the fasting glucose concentration was ≥126 mg/d, glucose concentration was ≥200 mg/dL in an oral 75-g 2-hour glucose tolerance test, HbA1c was ≥6.5%, and the use of glucose-lowering medication. A structured questionnaire was used to investigate regarding the use of glucose-lowering medication.

Insulin resistance was evaluated using HOMA-IR, VAI, LAP, and TyG index. The formulas for HOMA-IR and TyG index were as follows:

HOMA‑IR=fastinginsulin(μIU/mL)×fastingglucose(mmol/L)/22.5

$$\begin{array}{l} VAI=\left(waist\;circumference\;\left(cm\right)/\left(39.68+\left(1.88\times Body\;mass\;index\;\left(BMI\right)\right)\right)\right)\times (triglycerides\\ \left(mmol/L\right)/1.03)\times \left(1.31/HDL‑C\;\left(mmol/L\right)\right)\;for\;men,\;or\;(waist\;circumference\\ \left(cm\right)/\left(36.58+\left(1.89\times BMI\right)\right))\times \left(triglycerides\;\left(mmol/L\right)/0.81\right)\times \left(1.51/HDL‑C\;\left(mmol/L\right)\right)\;for\\ women, \end{array}$$

$$\begin{array}{l} LAP=\left(waist\;circumference\;\left(cm\right)-65\right)\times \left(triglycerides\;\left(mmol/L\right)\right)\;for\;men,\;or\;(waist\\ circumference\;\left(cm\right)-58)\times \left(triglycerides\;\left(mmol/L\right)\right)\;for\;women \end{array}$$

TyGindex=Ln(fastingglucose(mg/dL)×triglycerides(mg/dL))/2.

### Statistical analysis

Summary statistics are presented as mean and standard deviation (SD) or prevalence (%). The values of each surrogate measure for insulin sensitivity were presented by the 10^th^, 25^th^, 50^th^, 75^th^, and 90^th^ percentile. We analyzed each measure of insulin resistance using receiver operating characteristic (ROC) curve to estimate the predictive ability for the development of DM in 10 years. We performed the de Long’s test to identify which surrogate measures for insulin resistance were significantly superior. The cut-off value of each surrogate measure was determined as the value with the highest Youden index score in the specificity dominant area. Multivariate Cox proportional hazards regression models were constructed to evaluate the hazards ratio (HR) and 95% confidence interval [CI] for DM. Follow-up duration was calculated as the time from the first anthropometric and clinical measures to either the date of development of DM or the end of follow-up (December 31, 2012). In addition, OR for DM according to the continuous value of each measure was analyzed using restricted cubic spline splits with five knots.

Analyses were carried out using SPSS, version 25.0 (IBM, Armonk, NY, USA) and the statistical package R (version 3.3.2, R Foundation for Statistical Computing). The significance levels were set at 0.05.

### Ethics statement

The protocol of the study was approved by the institutional review board of Kangnam Sacred Heart Hospital (IRB No. HKS 2017-07-007), and all participants gave written informed consent. All participants’ records were anonymized before being accessed by the authors, and all methods were carried out in accordance with the approved guidelines and regulations.

## Results

### Baseline characteristics

Overall, data from 7,643 participants were assessed (3,603 males and 4,040 females). Among them, 17.1% (1,306) had newly diagnosed DM during the 10-year follow-up period. Table 1 summarizes baseline anthropometric, clinical, and biochemical characteristics of the participants.

**Table 1:** Baseline characteristics of participants according to incident DM.

Characteristics	Total participants (N = 7,643)
Age, years	51.7 ± 8.8
Male sex, n (%)	3,603 (47.1)
Smoking, n (%)	3,044 (40.3)
BMI, Kg/m^2^	24.4 ± 3.1
Energy intake, Kcal/day	1,967.7 ± 720.8
Physical activity*, n (%)	4,649 (62.6)
Hypertension, n (%)	2,271 (29.7%)
Systolic BP, mmHg	120.5 ± 17.9
Diastolic BP, mmHg	79.9 ± 11.4
HbA1c, %	5.5 ± 0.3
Fasting glucose, mg/dL	82.7 ± 8.5
Fasting insulin, μIU/mL	7.6 ± 4.8
Total cholesterol, mg/dL	189.6 ± 34.2
HDL Cholesterol, mg/dL	44.9 ± 10.0
Triglycerides, mg/dL	154.8 ± 94.0
HOMA-IR	1.6 ± 1.0
VAI	2.5 ± 1.9
LAP	38.5 ± 31.9
TyG index	4.7 ± 0.2

Data were presented as means ± SD or number (%)

* Participants who engaged in physical activity for at least 30 minutes per day

Abbreviations: BMI; body mass index; BP, blood pressure; HbA1c; hemoglobin A1c; HDL; high-density lipoprotein; HOMA-IR: homeostasis model assessment-insulin resistance; TyG index; triglycerides and glucose index

The distribution of surrogate measures for insulin resistance at baseline examination are summarized in Table 2.

**Table 2:** Distribution of surrogate measures for insulin resistance at baseline examination.

Indirect index	Percentile				
	10^th^	25^th^	50^th^	75^th^	90^th^
HOMA-IR	0.64	1.03	1.40	1.92	2.48
VAI	0.97	1.34	2.00	3.05	4.67
LAP	10.7	17.9	30.2	49.7	75.7
TyG index	4.37	4.49	4.64	4.81	4.99

Abbreviations: HOMA-IR: homeostasis model assessment-insulin resistance; VAI: visceral adiposity index; LAP: lipid accumulation product; TyG index: triglycerides and glucose index

### Cut-off values of surrogate measures for insulin resistance

**Fig 2:**
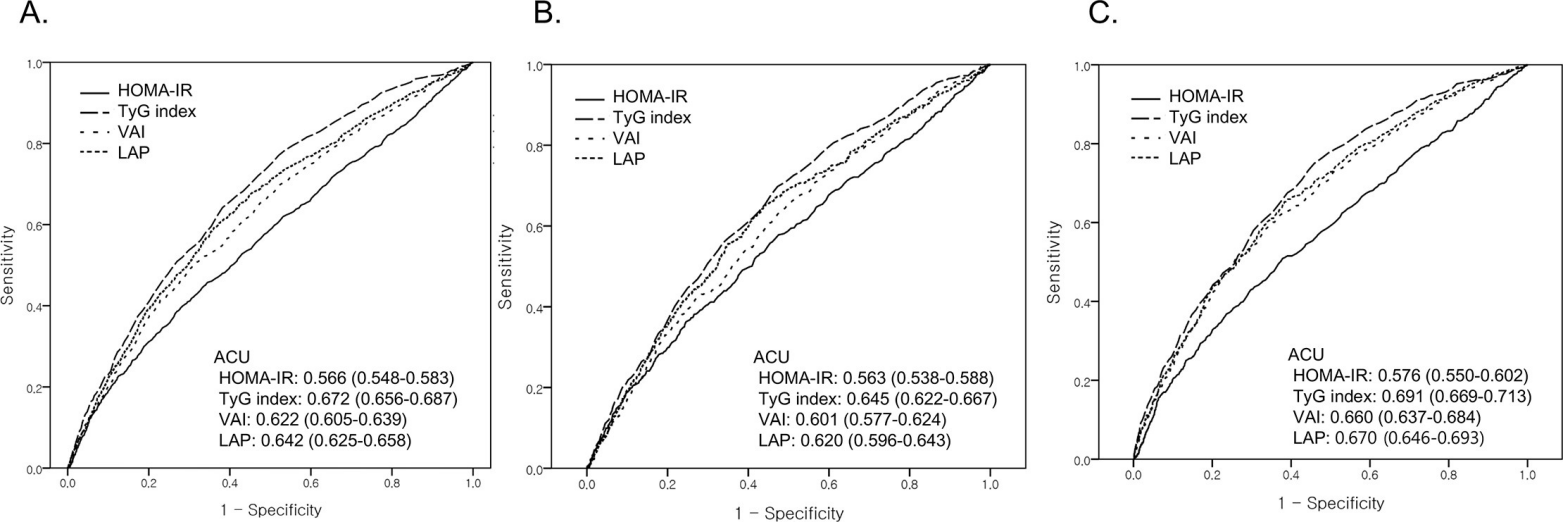
ROC curves of incident diabetes mellitus in 10 years based on each surrogate measure for insulin resistance. **A.** Total; **B.** Men; **C.** Women.

The ROC for newly developed DM in 10 years according to each measure is presented in Fig 2. The AUC was 0.566 (95% CI, 0.548–0.583) for HOMA-IR, 0.622 (95% CI, 0.605–0.639) for VAI, 0.642 (95% CI, 0.625–0.658) for LAP, and 0.672 (95% CI, 0.656–0.687) for TyG index. The AUC of TyG index was significantly higher than that of HOMA-IR, VAI, and LAP (p < 0.001). The AUC of VAI was similar with that of LAP (p = 0.115) while higher than that of HOMA-IR (p value < 0.001). The AUC of each measure was higher in women than in men.

**Fig 3:**
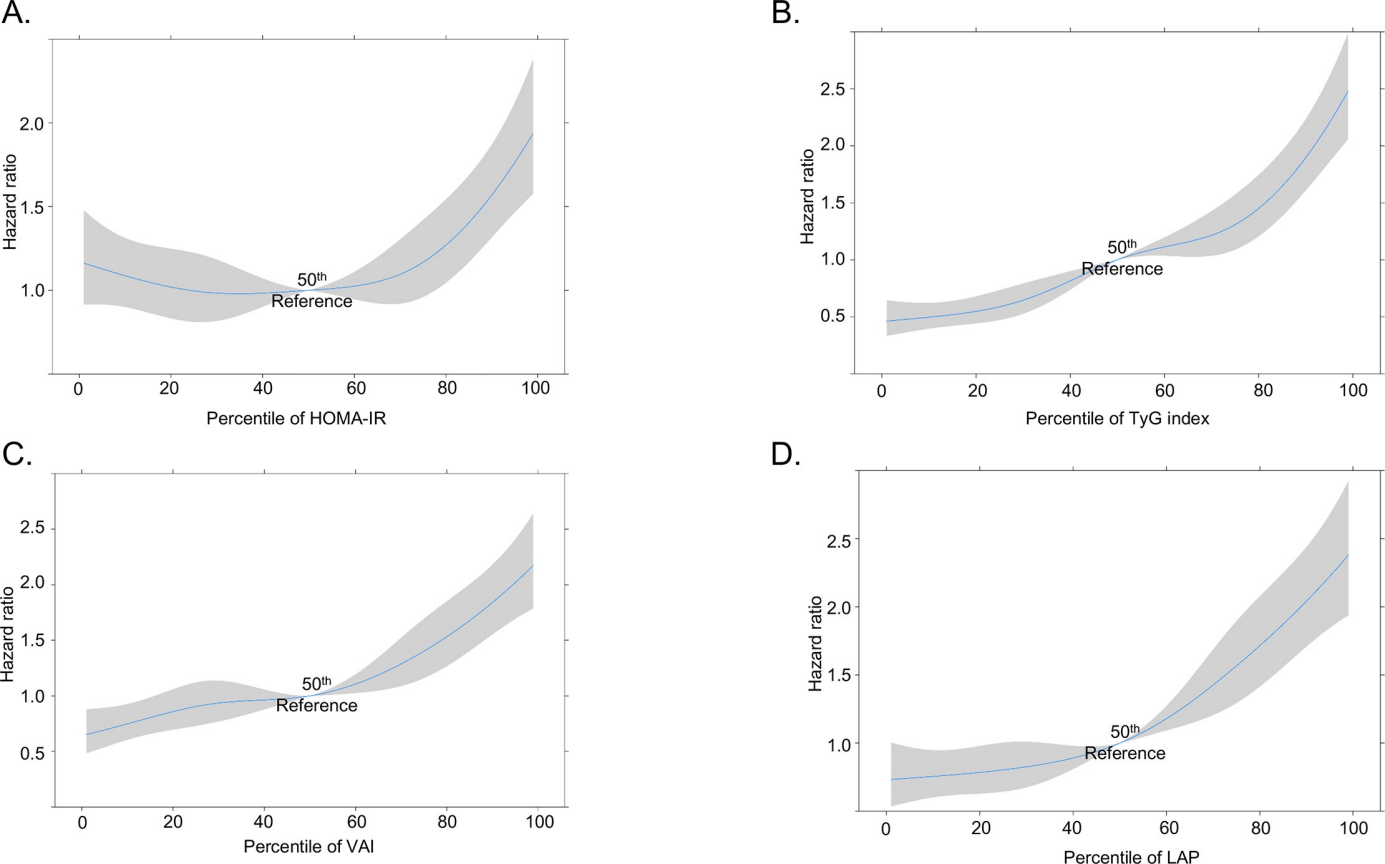
The odds ratio for DM according to the percentile of each surrogated measure. A. HOMA-IR; B. VAI; C. LAP; D. TyG index Abbreviations: HOMA-IR: homeostasis model assessment-insulin resistance; VAI: visceral adiposity index; LAP: lipid accumulation product; TyG index: triglycerides and glucose index.

The cut-off values with their corresponding sensitivity, specificity, and HR are summarized in **Table 3**. In the restricted cubic spline regression, each surrogate measure showed a dose-dependent relationship with the risk of DM (Fig 3).

**Table 3:** Cut-off values with their corresponding sensitivity, specificity, and hazard ratio (HR).

Surrogate measures	Cut-off value	Sensitivity	Specificity	HR (95% CI)*
HOMA-IR	1.83	38.6	73.1	1.41 (1.25–1.59)
VAI	2.54	50.4	68.8	1.75 (1.55–1.96)
LAP	36.6	59.2	63.9	1.87 (1.64–2.14)
TyG index	4.69	62.1	63.1	2.17 (1.92–2.45)

Abbreviations: HOMA-IR: homeostasis model assessment-insulin resistance; TyG index; triglycerides and glucose index; HR: hazard ratio

*Adjusted for age, sex, BMI, smoking and hypertension, physical activity, energy intake

## Discussion

In this community-based prospective cohort, we confirmed that TyG index was a better predictor for DM compared with VAI, LAP and HOMA-IR. VAI and LAP showed modest predictability for DM while HOMA-IR scarcely predicted DM.

HOMA-IR is a well-known robust tool for the assessment of insulin resistance and is associated with the development of DM [14–16]. The present study shows dose dependent association between HOMA-IR and the risk of DM. However, the AUC value was much lower than those in previous studies. According to studies conducted in Iran, China, and Korea, the AUC value of HOMA-IR was approximately 0.7–0.8 [17–19]. Interestingly, a significant decline of the diagnostic performance of HOMA-IR for type 2 DM was observed with aging [19]. Several previous studies showed a decline in the AUC of HOMA-IR for metabolic syndrome in elderly individuals as well [20, 21]. Considering that the participants of our study were older than those of previous studies, such differences might arise from age-related effects.

It is important to estimate a valid cut-off value for the clinical use of HOMA-IR. Although a number of studies suggested the cut-off values, there is great variability. In several population-based studies, the cut-off values of HOMA-IR were made based on the percentile criterion (75 -90^th^ percentile according to studies) of values in the general population [22–24]. However, considering the distribution of HOMA-IR varied according to participants’ demographic characteristics such as age, sex and race, it is difficult to estimate the optimal cut-off value with the percentile criterion. For example, the 75th percentile of HOMA-IR was 2.53 in healthy Koreans, while 1.6 in healthy Iranians, 2.0 in healthy Swedish men and 3.8 in French men [20, 22–24]. In addition, it is unclear whether the proposed cutoff values of HOMA-IR based on the percentile criterion could predict clinically relevant outcomes [15, 20, 21].

In order to resolve such doubts, several studies were conducted to determine the valid cut-off value of HOMA-IR for predicting the development of DM. Despite the fact that DM develops over a long period of time, most of the studies were cross-sectional and may have contained various confounding factors [25–27]. Limited data regarding HOMA-IR cut-off values were obtained from longitudinal studies. Ghasemi et al. suggested that the cut-off value of HOMA-IR was 2.17 (sensitivity 50%, specificity 76.7%) for males and 1.85 (sensitivity 75.9%, specificity 58.3%) for females [17]. According to Lee et al., the cut-off value of HOMA-IR was 1.97 (sensitivity 65.5%, specificity 82.9%) [18]. The results obtained in our study are consistent with those obtained in these studies. However, considering the low sensitivity of HOMA-IR for DM in the present study, this cut-off value has limitations for the application to clinical settings.

One significant drawback of HOMA-IR is that a standard assay for measurement of fasting insulin is absent. To overcome this, insulin-free equations for estimating insulin resistance have been developed. One well-known useful insulin-free surrogate measure is the triglycerides and glucose (TyG) index [10]. It is a more simple and inexpensive method compared with insulin-based surrogate measures. TyG is well correlated with the gold standard methods for insulin resistance such as HIEG clamp or MMAMG [10, 28]. Moreover, there was a modest correlation between the TyG index and insulin stimulated glucose uptake during insulin suppression testing [29]. Several population-based studies demonstrated that high TyG index was associated with DM, hypertension, nonalcoholic fatty liver disease, and atherosclerosis [30–35]. However, there have been few studies to estimate the cut-off value of TyG index for predicting development of DM. Guerrero-Romero et al. suggested that the best value of the TyG index for diagnosis of insulin resistance was 4.68 using the HIEG clamp test with a small sample size; this cut-off value is similar to our findings [10]. We suggest our results could support the cut-off value from the study of Guerrero-Romero et al. To the best of our knowledge, this is the first study to estimate a valid cut-off value of TyG index using data from a population-based longitudinal study.

Notably, we found that TyG index had better predictive power for development of DM, compared with HOMA-IR. The correlation between HIEG clamp test and TyG index is known to be comparable with the correlation between HIEG clamp test and HOMA-IR [28, 36]. However, a direct comparison between TyG index and HOMA-IR regarding their ability to predict DM development has not performed yet. Therefore, another significance of the present study is providing evidence of clinical usefulness of TyG index for identifying individuals at risk for DM. Regarding low cost and universal use of blood glucose and triglycerides tests, the TyG index can be a good supplementary test measure for insulin resistance.

Interestingly, the AUC of the surrogate measures differed by sex. This result is consistent with those of a previous study [17, 19]. Considering that estrogens promote peripheral fat storage, whereas androgens promote the accumulation of visceral abdominal fat, the alteration of sex hormone might affect the insulin resistance and the diagnostic performance of each measure for type 2 DM [37].

The main strength of this study was the data source, the Ansung-Ansan Cohort, which is a long-term (10-year follow-up), community-based cohort. Despite this strength, the present study has some limitations. First, because this study was performed among Korean adults, the result might not be applicable to other ethnicities. Second, the study lacks in directly comparing the surrogate measure and gold standard methods for insulin resistance such as HIEG clamp or MMAMG. Further studies are necessary for this issue.

In conclusion, TyG index was better predictor for DM compared with HOMA-IR. VAI and LAP showed modest predictability for DM. TyG index could be used as a simple and supplementary method to identify individuals at risk for insulin resistance and DM development.

## References

[1] Kwak SH, Park KS. Pathophysiology of Type 2 Diabetes in Koreans. Endocrinol Metab (Seoul). 2018;33:9–16. 29589384 10.3803/EnM.2018.33.1.9 PMC5874201

[2] Samuel VT, Shulman GI. Mechanisms for insulin resistance: common threads and missing links. Cell. 2012;148:852–71. 10.1016/j.cell.2012.02.017 22385956 PMC3294420

[3] Ascaso JF, Pardo S, Real JT, Lorente RI, Priego A, Carmena R. Diagnosing insulin resistance by simple quantitative methods in subjects with normal glucose metabolism. Diabetes Care. 2003;26:3320–5. 14633821 10.2337/diacare.26.12.3320

[4] Hanefeld M, The metabolic syndrome: roots, myths, and facts In: Hanefeld M, Leonhardt W, editors, The Metabolic Syndrome, Jena: Gustav Fischer;1997,p.13–24.

[5] Defronzo R, Tobin J, Andres R. Glucose clamp technique: a method for quantifying insulin secretion and resistance. Am J physiol. 1979;237:214–23. 10.1152/ajpendo.1979.237.3.E214 382871

[6] Bergman RN, Prager R, Volund A, Olefsky JM. Equivalence of insulin sensitivity index in man derived by the minimal model method and the euglycemic glucose clamp. J Clin Invest. 1987;79:790–800. 10.1172/JCI112886 3546379 PMC424201

[7] Greenfield MS, Doberne L, Kraemer F, Tobey T, Reaven G. Assessment of insulin resistance with insulin suppression test and euglycemic clamp. Diabetes. 1981;30:387–92. 7014307 10.2337/diab.30.5.387

[8] Matthews DR, Hosker JP, Rudenski AS, Naylor BA, Treacher DF, Turner RC. Homeostasis model assessment: insulin resistance and b cell function from fasting plasma glucose and insulin concentration in man. Diabetologia. 1985;28:412–9. 3899825 10.1007/BF00280883

[9] Katz A, Nambi SS, Mather K, Baron AD, Follmann DA, Sullivan G, et al Quantitative insulin sensitivity check index: a simple accurate method for assessing insulin sensitivity in humans. J Clin Endocrinol Metab. 2000;85:2402–10. 10.1210/jcem.85.7.6661 10902785

[10] Guerrero-Romero F, Simental-Mendía LE, González-Ortiz M, Martínez-Abundis E, Ramos-Zavala MG, Hernández-González SO, et al The product of triglycerides and glucose, a simple measure of insulin sensitivity. Comparison with the euglycemic-hyperinsulinemic clamp. J Clin Endocrinol Metab. 2010;95:3347–51. 10.1210/jc.2010-0288 20484475

[11] Mazidi M, Kengne AP, Katsiki N, Mikhailidis DP, Banach M. Lipid accumulation product and triglycerides/glucose index are useful predictors of insulin resistance. J Diabetes Complications. 2018;32:266–70 10.1016/j.jdiacomp.2017.10.007 29395839

[12] Kim Y, Han BG; KoGES group. Cohort Profile: The Korean Genome and Epidemiology Study (KoGES) Consortium. Int J Epidemiol. 2017;46:e20 10.1093/ije/dyv316 27085081 PMC5837648

[13] Korean Statistical Information Service. Available from: http://kosis.kr. Accessed 5 May 2018.

[14] Lann D, LeRoith D. Insulin resistance as the underlying cause for the metabolic syndrome. Med Clin North Am. 2007;91:1063–77. 10.1016/j.mcna.2007.06.012 17964909

[15] Antuna-Puente B, Disse E, Rabasa-Lhoret R, Laville M, Capeau J, Bastard JP. How can we measure insulin sensitivity/resistance? Diabetes Metab. 2011;37:179–88. 10.1016/j.diabet.2011.01.002 21435930

[16] Song YS, Hwang YC, Ahn HY, Park CY. Comparison of the Usefulness of the Updated Homeostasis Model Assessment (HOMA2) with the Original HOMA1 in the Prediction of Type 2 Diabetes Mellitus in Koreans. Diabetes Metab J. 2016;40:318–25. 10.4093/dmj.2016.40.4.318 27273908 PMC4995187

[17] Ghasemi A, Tohidi M, Derakhshan A, Hasheminia M, Azizi F, Hadaegh F. Cut-off points of homeostasis model assessment of insulin resistance, beta-cell function, and fasting serum insulin to identify future type 2 diabetes: Tehran Lipid and Glucose Study. Acta Diabetol. 2015;52:905–15 10.1007/s00592-015-0730-3 25794879

[18] Lee CH, Shih AZ, Woo YC, Fong CH, Leung OY, Janus E, et al Optimal Cut-Offs of Homeostasis Model Assessment of Insulin Resistance (HOMA-IR) to Identify Dysglycemia and Type 2 Diabetes Mellitus: A 15-Year Prospective Study in Chinese. PLoS One. 2016;11:e0163424 10.1371/journal.pone.0163424 27658115 PMC5033570

[19] Baek JH, Kim H, Kim KY, Jung J. Insulin Resistance and the Risk of Diabetes and Dysglycemia in Korean General Adult Population. Diabetes Metab J. 2018;42:296–307 10.4093/dmj.2017.0106 29885105 PMC6107354

[20] Moon S, Park JH, Jang EJ, Park YK, Yu JM, Park JS, et al The Cut-off Values of Surrogate Measures for Insulin Sensitivity in a Healthy Population in Korea according to the Korean National Health and Nutrition Examination Survey (KNHANES) 2007–2010. J Korean Med Sci 2018;33:e197 10.3346/jkms.2018.33.e197 30008630 PMC6041480

[21] Gayoso-Diz P, Otero-González A, Rodriguez-Alvarez MX, Gude F, García F, De Francisco A, et al Insulin resistance (HOMA-IR) cut-off values and the metabolic syndrome in a general adult population: effect of gender and age: EPIRCE cross-sectional study. BMC Endocr Disord 2013;13:47 10.1186/1472-6823-13-47 24131857 PMC4016563

[22] Hedblad B, Nilsson P, Janzon L, Berglund G. Relation between insulin resistance and carotid intima-media thickness and stenosis in non-diabetic subjects. Results from a cross-sectional study in Malmo, Sweden. Diabet Med. 2000;17: 299–307. 10821297 10.1046/j.1464-5491.2000.00280.x

[23] Esteghamati A, Ashraf H, Esteghamati AR, Meysamie A, Khalizadeh O, Nakhjavani M, et al Optimal threshold of homeostasis model assessment for insulin resistance in an Iranian population: the implication of metabolic syndrome to detect insulin resistance. Diabetes Res Clin Pract. 2009;84:279–87. 10.1016/j.diabres.2009.03.005 19359063

[24] Marques-Vidal P, Mazoyer E, Bongard V, Gourdy P, Ruidavets JB, Drouet L, et al Prevalence of insulin resistance syndrome in Southwestern France and its relationship with inflammatory and haemostatic markers. Diabetes Care. 2002;25:1371–7. 12145237 10.2337/diacare.25.8.1371

[25] Ryu S, Sung KC, Chang Y, Lee WY, Rhee EJ. Spectrum of insulin sensitivity in the Korean population. Metabolism. 2005;54:1644–51. 10.1016/j.metabol.2005.06.014 16311099

[26] Lee S, Choi S, Kim HJ, Chung YS, Lee KW, Lee HC, et al Cutoff values of surrogate measures of insulin resistance for metabolic syndrome in Korean non-diabetic adults. J Korean Med Sci. 2006;21:695–700. 10.3346/jkms.2006.21.4.695 16891815 PMC2729893

[27] Tomé MA, Botana MA, Cadarso-Suarez C, Rego-Irateta A, Fernandez-Mariño A, Mato JA, et al Prevalence of metabolic syndrome in Galicia (NW Spain) on four alternative definitions and association with insulina resistance. J Endocrinol Invest. 2009;32:505–11. 10.3275/6102 19465797

[28] Vasques AC, Novaes FS, de Oliveira Mda S, Souza JR, Yamanaka A, Pareja JC, et al TyG index performs better than HOMA in a Brazilian population: a hyperglycemic clamp validated study. Diabetes Res Clin Pract. 2011;93:e98–e100. 10.1016/j.diabres.2011.05.030 21665314

[29] Abbasi F, Reaven GM. Comparison of two methods using plasma triglyceride concentration as a surrogate estimate of insulin action in nondiabetic subjects: triglycerides × glucose versus triglyceride/high-density lipoprotein cholesterol. Metabolism. 2011;60:1673–6. 10.1016/j.metabol.2011.04.006 21632070

[30] Simental-Mendía LE, Rodríguez-Morán M, Guerrero-Romero F. The product of fasting glucose and triglycerides as surrogate for identifying insulin resistance in apparently healthy subjects. Metab Syndr Relat Disord. 2008;6:299–304. 10.1089/met.2008.0034 19067533

[31] Lee SH, Kwon HS, Park YM, Ha HS, Jeong SH, Yang HK, et al Predicting the development of diabetes using the product of triglycerides and glucose: the Chungju Metabolic Disease Cohort (CMC) study. PLoS One. 2014;9:e90430 10.1371/journal.pone.0090430 24587359 PMC3938726

[32] Kim MK, Ahn CW, Kang S, Nam JS, Kim KR, Park JS. Relationship between the triglyceride glucose index and coronary artery calcification in Korean adults. Cardiovasc Diabetol. 2017;16:108 10.1186/s12933-017-0589-4 28830471 PMC5568209

[33] Lee SB, Ahn CW, Lee BK, Kang S, Nam JS, You JH, et al Association between triglyceride glucose index and arterial stiffness in Korean adults. Cardiovasc Diabetol. 2018;17:41 10.1186/s12933-018-0692-1 29562908 PMC5863385

[34] Zhang S, Du T, Zhang J, Lu H, Lin X, Xie J, et al The triglyceride and glucose index (TyG) is an effective biomarker to identify nonalcoholic fatty liver disease. Lipids Health Dis. 2017;16:15 10.1186/s12944-017-0409-6 28103934 PMC5248473

[35] Zheng R, Mao Y. Triglyceride and glucose (TyG) index as a predictor of incident hypertension: a 9-year longitudinal population-based study. Lipids Health Dis. 2017;16:175 10.1186/s12944-017-0562-y 28903774 PMC5598027

[36] Guerrero-Romero F, Villalobos-Molina R, Jiménez-Flores JR, Simental-Mendia LE, Méndez-Cruz R, Murguía-Romero M, et al Fasting Triglycerides and Glucose Index as a Diagnostic Test for Insulin Resistance in Young Adults. Arch Med Res. 2016;47:382–7. 10.1016/j.arcmed.2016.08.012 27751372

[37] Stefanska A, Bergmann K, Sypniewska G. Metabolic syndrome and menopause: pathophysiology, clinical and diagnostic significance. Adv Clin Chem 2015;72:1–75. 10.1016/bs.acc.2015.07.001 26471080

